# Comparison of trace element concentration in bone and intervertebral disc tissue by atomic absorption spectrometry techniques

**DOI:** 10.1186/s13018-014-0099-y

**Published:** 2014-10-25

**Authors:** Łukasz Kubaszewski, Anetta Zioła-Frankowska, Marcin Frankowski, Piotr Rogala, Zuzanna Gasik, Jacek Kaczmarczyk, Andrzej Nowakowski, Mikolaj Dabrowski, Wojciech Labedz, Grzegorz Miękisiak, Robert Gasik

**Affiliations:** Department of Orthopedics and Traumatology, W. Dega University Hospital, University of Medical Science Poznan, 28 Czerwca 1956r St., Poznań, 61-545 Poland; Department of Water and Soil Analysis, Faculty of Chemistry, Adam Mickiewicz University in Poznań, Umultowska 89b, Poznań, 61-614 Poland; Department of Spine Surgery, Oncologic Orthopaedics and Traumatology, W. Dega University Hospital, University of Medical Science Poznan, 28 Czerwca 1956r St., Poznań, 61-545 Poland; Clinic and Polyclinic of Neuroorthopedic and Neurology, Institute of Rheumatology, Warsaw, Spartańska 1, Warsaw, 02-637 Poland; Department of Neurosurgery, Specialist Medical Center, Polanica-Zdroj, Poland

**Keywords:** Bone, Intervertebral disc, Trace element

## Abstract

**Background:**

Trace element (TE) analysis in human tissue has the dual purpose of assessing environmental pollution and metabolism. In literature, bone TE analysis is common, but studies in intervertebral disc (IVD) tissue are lacking. The aim of the study was evaluation of the difference of TE concentration in intervertebral disc and bone in patients with degenerative changes. The comparison of the tissues differing in metabolism, blood perfusion, or separateness from adjoining tissues but playing similar biomechanical role and presenting some common morphological traits may shed new light on metabolism nuances, degenerative process, as well as accumulation potential of IVD in respect to bone.

**Methods:**

In the study, we analyzed two types of samples: intervertebral disc (*n* =30, from 22 patients operated due to degenerative disc disease) and femoral bone (*n* =26, separately femoral head and neck, from 26 patients, acquired in total hip arthroplasty procedure in course of idiopathic osteoarthritis of the hip joint). In the samples we analyzed, with atomic absorption spectrometry, the concentrations of Pb, Ni, Mo, Cu, Mg, and Zn.

**Results:**

The element concentrations identified in bone are comparable to those presented in the literature. In the case of Pb, Ni, Mo, Mg, and Zn, the concentration in the bone was 2 to 25.8 times higher than that observed in the disc. Only the Cu concentration was higher in disc tissue than in bone. In disc tissue, fewer samples had TE concentrations below the detection threshold.

We found significant differences in TE profiles in the compared tissues.

**Conclusions:**

The results show that the disc could serve as a more stable compartment for evaluating TE concentration, especially for TEs that are environmentally related.

## Background

Trace element (TE) analysis in biota has a twofold purpose of monitoring environmental exposure to the pollutants and, secondarily, of indirect analysis of metabolism-related issues. The human body can be divided into several compartments with respect to element turnover. The most unstable compartments are serum, urine, and cerebrospinal fluid. Analysis of these tissues is useful for short periods of exposure or organismal response to specific metabolic impulses. In contrast is bone tissue, which is regarded as a TE repository that reflects turnover in the whole organism [1]. The biological half life of heavy metals in bones may be up to 30 years and reflect up to 90% of the whole body content [2], possibly because of the solid structure of the bone tissue along with optimal blood perfusion.

A distinct compartment of the human body is the intervertebral disc. Transformation during maturation causes loss of vascularity within the first decade of life [3] and is followed by metabolism transformation to withstand low oxygen concentration and a highly acidic environment because of waste product concentration. IVD morphology is characterized by a dominating extracellular matrix seeded with a low density of chondrocyte-like cell clusters responsible for production and control of matrix turnover. Domination of the extracellular matrix is similar in bone and IVD tissue. Dissimilarities are related to blood perfusion that reflects metabolism. Such separateness of the IVD tissue makes it of interest for TE concentration analysis.

In this study, we evaluate the trace elements concentration in intervertebral disc tissue and femoral bone in patient with degenerative changes. There is high disproportion in a number of studies of two examined tissues. Only few papers present the concentration and accumulation potential for selected TEs in IVD [4,5], no comparison analysis between IVD and bone tissue has been presented.

The advantages of the analysis were comparison of the tissues that substantially differ in metabolism, blood perfusion, and separateness form adjoining tissues and organism. Nevertheless, tissues are similar, for biomechanical function and morphology with dominating extracellular matrix. As the pilot study, we have decided to choose the example elements from three groups: essential, potentially essential, and toxic to recognize the distribution differences in both tissues. Such analysis not only sheds a new light on the metabolism especially of the intervertebral disc but also evaluates the accumulation potential of the IVD in respect to the bone tissue. Addition value of the study was performing the analysis with the same methodology by the same laboratory.

Among the available analytical techniques, the GF-AAS analytical technique is better for determination of elements in biological samples because of, e.g., only few spectral interferences, good limits of detection, small sample volume, and low analysis costs. Besides for determination of single structural elements, (e.g., Mg and Zn) flame atomic absorption spectrometry is better because of the low cost. Also the GF-AAS technique is the most commonly used technique for the analysis of mineral and trace elements in biological samples, e.g., Pb in bones samples [6]; Cr, Cd, Mn, Ni, and Pb in whole blood, urine, saliva, and axillary hair [7]; and Al in bones [8].

Another analytical techniques used in analysis of metals in biological samples are, e.g., X-ray fluorescence (Pb and Sr in bones) [9,10]; Prompt gamma neutron activation (Cd in liver and kidney) neutron activation analysis (Al in bones) [11], ICP-AES (Cu, Co, Cr, Y, Yb, and Bi in biological samples) [12]; ICP-MS (trace elements in human urine) [13]; and HPLC-ICP-AES (Cu, Cd, and Zn in human liver) [14].

The aim of the study was evaluation the differences of the trace elements concentration in intervertebral disc tissue and femoral bone in patient with degenerative changes with graphite furnace atomic absorption spectrometry (GF-AAS) technique.

## Methods

This analysis involved two groups: patients with degenerative disc disease (DDD) and patients with idiopathic osteoarthritis of the hip joint (OA).

### DDD group

Intervertebral discs from 22 patients were obtained during a surgical procedure. Twelve specimens (6 patients) were from the cervical spine and 18 (16 patients) were taken from the lumbar spine. The indication for the operation was degenerative disc disease with neurological changes in clinical examination: local neck or back pain with radicular symptoms. In the cervical spine, the discectomy was performed with an anterior approach with the removal of the intervertebral disc tissue followed with interbody fusion. In the lumbar spine, the intervertebral disc was approached from posterior. After tissue removal in seven cases, the interbody fusion with transpedicular stabilization was performed to restore the stability of the motion segment. During the acquisition of the biological material, only the intervertebral disc was taken to the analysis without the parts of the vertebral end-plate removed in the preparation process for interbody fusion. The samples were frozen in −20°C.

### OA group

This study included the material of 26 femoral bone fragments from 26 patients, acquired during total hip arthroplasty. During the surgical procedure, the proximal part of the femur was resected with a motor saw. After resection, the sample was cleaned from adjoining soft tissues, i.e., joint capsule or muscles and frozen in −20°C. The indication for the procedure was idiopathic osteoarthrosis of the hip joint. A separate analysis was performed for two anatomic regions of each type of resected fragment: the femoral head and femoral neck.

### Patient data and sample analysis

All patients were interviewed using a questionnaire to collect data on demography, health status, and occupational heavy metal exposure. In the interview, no patients had knowledge of inadvertent exposure to heavy metal pollution.

In all samples, the levels of Pb, Ni, Mo, Cu, Mg, and Zn were evaluated. In OA samples, TEs were evaluated separately for the femoral neck and head.

The frozen intervertebral disc samples were freeze-dried using a Lyovac lyophilizer GT2e (Steris, Germany) for 24 h (drying pressure of *p* = 6.5 × 10^−1^ mbar, ambient temperature under vacuum—approximately −55°C). After drying of the IVD samples, the femoral head and femoral neck samples were weighed, and nitric acid (Suprapur, Merck, Germany) was added to obtain a dilution factor of 10 (range of sample weight: 0.111–0.489 g dry weight [dw]). The prepared samples were allowed to stand overnight to slow mineralization. Samples then were mineralized in a microwave oven (Mars Xpress 5, USA) based on modified 3051 EPA method [15]. TE concentrations were calculated for the dry weight of the disc.

The TE concentrations were determined in three replications using an atomic absorption spectrometer (AA 7000, Shimadzu, Japan) with graphite furnace atomization (GF-AAS). The percent relative standard deviation (%RSD) for the GF-AAS analytical technique did not exceed 5%. The concentrations of Mg and Zn were determined in three replications using the AA 7000 (Shimadzu, Japan) with flame atomization (F-AAS). The %RSD for the F-AAS analytical technique did not exceed 7%.

In the analysis, the following values for the traits were derived: mean, median, maximum and minimum, and standard deviation (SD).

The Shapiro-Wilk test was used to confirm the normal distribution of the analyzed parameters. In case of normal distribution, Student’s *t*-test for independent samples was used, if sample values were non-normally distributed, nonparametric statistical test was used (Mann-Whitney test). To evaluate the difference between groups DDD and OA in respect to sex, the Chi^2^ test (*p* =1,0) was applied. Spearman rank correlation test was used for age correlation with TE concentration. *p* values <0.05 were considered statistically significant.

### Ethics statement

Ethical considerations were in agreement with the Helsinki Declaration. In all cases, patients were informed about the aim of study and gave written consent for participation in the study and for data publication. The use of tissue in the investigations was approved by the Bioethics Committee of the Institute of Rheumatology, Warsaw, on May 31, 2012, and Bioethics Committee of University of Medical Sciences, Poznan, reference nos. 406/13 and 172/14.

## Results and discussion

The TE values for both groups are presented in Table 1. Among patients with DDD, the average age was 47.6 years (range 28–64; SD 8.8). Among patients with OA, the average age was 57.8 (range 30–64; SD 7.2). Among DDD patients, males constituted the majority (54.5%) of the group; in OA, the majority also were males (61.5%).

**Table 1:** **Characteristics of trace element concentrations in the analyzed groups**

	**Element**					
	**Pb**	**Ni**	**Mo**	**Cu**	**Mg**	**Zn**
	**μg kg** ^**−1**^ **dw**	**μg kg** ^**−1**^ **dw**	**μg kg** ^**−1**^ **dw**	**mg kg** ^**−1**^ **dw**	**mg kg** ^**−1**^ **dw**	**mg kg** ^**−1**^ **dw**
DDD						
Min–max	61.47–2,233	25.48–444.2	20.02–143.2	0.971–6.091	182.6–2,132	10.56–184.5
Mean	686.7	215.4	54.33	2.71	800.1	39.61
Median	510.9	169.9	47.75	2.504	624.5	31.99
SD	565.6	132.1	27.53	1.352	525.5	35.95
OA—femoral neck						
Min–max	1,266–5,090	294.0–3,602	360.0–1,900	0.550–2.660	1,163–2,243	52.67–108.2
Mean	2,898*	1,511*	1,244*	1.210^‡^	1,661*^†^	72.23
Median	3,227	1,139	1,425	1.117	1,617	67.71
SD	1,359	1,290	502.2	0.540	318.9	15.19
OA—femoral head						
Min–max	1,436–6,279	194.2–6,438	1,105–1,663	0.550–2.550	900.4–2,529	53.51–96.89
Mean	3,403*	1,467	1,376*	1.630*^‡^	1,458*^†^	74.40*
Median	3,416	838	1,338	1.760	1,413	73.50
SD	1,540	1,727	213.5	0.540	384.3	13.32

*Normal distribution, ^‡^statistically significant (Mann-Whitney test), ^†^statistically significant (Student’s *t*-test).

There were no statistically significant differences between two groups in respect to sex. Although the age ranges were similar in both groups, there was no ground to support the null hypothesis (*p* >0.05).

In the OA group, we confirmed the statistically significant higher concentration of the Ni in femoral neck in males. In femoral head group concentration of Zn, Cu, Ni, and Pb was higher in males, but the differences were not statistically significant. In the DDD group, we have found positive significant correlation of Pb concentration with age. No differences between sexes have been observed in respect to TE concentrations.

Stage of osteoarthritis in OA samples was moderate or severe. The cartilage of femoral head showed partial or total damage in the area of 50%–90% of the cartilage.

In the DDD group, TE concentrations were within detection limits in the vast majority of samples. Only in the case of Mo (five samples) and Ni (one sample) were concentrations below the level of detection (LOD). The percentages of undetectable TE were 16% and 3%, respectively.

In bone samples acquired from the femoral neck, Pb was undetected in 17 samples (68%), Ni in 19 (76%), Mo in 13 (52%), and Cu in 12 (48%). In bone samples acquired from the femoral head, Pb was undetected in 16 samples (64%), Ni in 12 (48%), Mo in 20 (80%), and Cu in 10 (40%). In the statistical analysis, those measurements were excluded.

The mean TE values were higher in bone (from twofold to almost 26-fold) in all cases except for Cu, which was up to 1.9 times higher (2.71 mg kg^−1^) in the disc compared to the femoral neck and head (1.21 and 1.63 mg kg^−1^, respectively). The minimal level of Cu detected in bone tissue was lower compared to IVD (0.55 vs 0.97 mg kg^−1^, respectively). In the DDD group, the maximum value of Cu significantly exceeded levels observed in both femoral neck and head. In one IVD sample, Cu levels reached 23.64 mg kg^−1^, which was excluded from the statistical analysis as an outlier. After exclusion of this measurement from the analysis, the maximum values for Cu in IVD and the femoral neck and head were 6.09, 2.56, and 2.55 mg kg^−1^, respectively. The SD for the disc tissue was more than double that of bone (1.35 vs 0.54 mg kg^−1^, respectively).

Our groups were derived from two studies, so sample selection targeted obtaining comparable groups in respect to age and sex. Generating groups that are matched for age can be a special challenge, particularly with a large age variance in the analyzed pathologies, as in this case. Furthermore, obviously, the disc and femoral bone cannot be collected from the same individuals. In our opinion, the selection of groups for this analysis was as optimal as possible given these limitations.

In some of our samples, the analyzed elements were below the LOD. We have not encountered such a situation in the literature evaluating TE in human tissue. In chemometric analysis, extensively used in spectrophotometric studies, samples for which the concentration is below the LOD are usually substituted with 0.5× LOD values [16]. However, we believe that presenting the data without this kind of substitution does not cause distortion and better reflects the clinical reality.

In both groups, there were samples with Mo and Ni concentrations below LOD. Otherwise, Mo and Ni were not detected in some samples of bone and IVD; in the latter case, the percentages were significantly lower than in bone. These elements are considered essential (Mo) or possibly essential (Ni) for humans [17].

Molybdenum is responsible for stabilizing oxidized nitrogen and occurs in organic molecules such as amino acids and carbohydrates in the form of MoO_4_^2−^ [18]. It is involved in xanthine oxidase synthesis, which is directly related to Mo content and affects protein synthesis and metabolism of purines and fats [17]. There are studies indicating the inverse relation of the Mo and Mg concentration that may be organ specific [19]. The element occurs in all human tissues in a range from 0.001 to 0.4 mg kg^−1^, with the lowest values for the blood and the highest for the kidneys and liver [17]. The average content in soft tissues (of the “reference man”) is <0.075 mg kg^−1^ and in skeleton <0.48 mg kg^−1^ [20]. Our study shows higher values of Mo in the bone, ranging from 0.360 to 1.9 mg kg^−1^. In the IVD, Mo concentration was approximately 25.8 times lower compared to bone and was in the “reference man” ranges for soft tissue.

In mammals, Ni is incorporated into superoxide dismutase, which uses divalent Zn [21]. Ni deficiency can induce some dysfunction in fat metabolism, but except for toxicity data, findings describing a potential metabolic role of the element are insufficient [17]. Studies indicate Ni supplementation to be bound with improved bone strength in birds [22]. On our study, Ni concentration in femoral neck was found to be related with male sex, where osteoporotic changes are observed later than in females. Brodziak-Dopierala et al. [23] estimated the Ni concentration in the femoral head at a medium level of 4.82 mg kg^-1^ (SD 10.74 mg kg^−1^) and the cartilage at 4.40 mg kg^−1^ (SD 7.38 mg kg^−1^). The average Ni content in human soft tissues is estimated at 0.088 mg kg^−1^ [17]. Its concentrations in soft human organs vary greatly, with the highest mean values for the lung (0.173 mg kg^−1^) and the lowest for the pancreas (0.034 mg kg^−1^) [17]. Our study showed a bone concentration of Ni to be lower than in Brodziak-Dopierala et al. (mean 1.46–1.51 vs 4.82 mg kg^−1^, respectively). Ni concentration in the IVD was still higher than reference values for soft tissues presented in the literature [23].

In the OA group, in up to 68% of the samples, the concentration of Pb was below the LOD. It is interesting that Pb levels below LOD were observed in bone tissue even as the mean value for the remainder of the samples was more than four times that of the disc tissue. Also only in the DDD group, we confirmed the significant correlation of the element with the age. Pb level is purely related to environmental pollution with no identified metabolic role. Additionally, it is considered highly toxic and is present in every human tissue in the range of <0.2 to 4.8 mg kg^−1^ [17]. The source can be food, water, or air. As an element, it is classified as a poor metal and a member of the carbon group. Pb influences heme synthesis by inhibiting porphobilinogen synthesis and ferrochelatase. It can lead to anemia by preventing formation of porphobilinogen and iron incorporation [24]. In neural tissue, Pb may be substituted as a calcium analog, interfering with ion channels during impulse conduction [17]. The lead exposure increases the risk of hip fracture in both males and females [25]. No data appear to be available regarding Pb function in the connective tissue that is characteristic for IVDs or Pb concentration in this tissue. Brodziak-Dopierala et al. estimated Pb concentration to be 3.75 mg kg^−1^ (SD 5.86 mg kg^−1^) [23]. Loska et al. identified lower levels with a mean of 1.35 mg kg^−1^ and SD of 0.68 mg kg^−1^ [26]. In our analysis, Pb bone levels were closer to one, in agreement with Brodziak-Dopierala et al. In humans, more than 90% of Pb accumulates in bones, and over 70% of that is found in the cortical bone [27]. The biological half-life of Pb in bones is estimated at about 30 years [2], and data related to connective tissue are lacking. None of the participants in our study reported specific exposure to environmental pollution; therefore, we have to assume a rather uniform exposure of our study groups. For this reason, we infer from our results that IVD is less accessible to environmental pollutants even as it presents a more stable concentration.

The next interesting finding comes from the Cu analysis. Cu is the only TE found at a higher concentration in IVD compared to bone. It was detected in some samples from the OA group while we observed its consistent presence in disc tissue. Cu is an essential element. In humans, it is part of ceruloplasmin, albumins, and amino acids and serves as a co-factor of lysyl oxidase, which is essential in cross-linking of collagen and elastin fibers [17]. Cu also acts together with iron in oxidation–reduction reactions and hemoglobin synthesis and is found in cytochrome c oxidase and superoxide dismutases [28]. In addition, this element influences polypeptide formation [29]. In serum level analysis, copper concentration, unlike calcium, does not show correlation with degenerative disc disease [30]. Also it does not show correlation with osteopenia or osteoporosis in postmenopausal women [31,32].

The range of Cu in human tissues is between 0.7 and 7.8 mg kg^−1^, with the lowest concentration in muscles and the highest in liver. Mean Cu level in tissues of the “reference man” is considered to be about 1 mg kg^−1^ [20], and the mean Cu concentration documented in the bone is from 0.62 [9] to 0.8 mg kg^−1^ dw [33]. Lanocha et al. found that Cu concentration in bone was not much different from that in cartilage, with an average concentration of 0.79 mg kg^−1^ dw, ranging between 0.20 and 1.78 mg kg^−1^ [33]. In our study, the Cu bone concentration was higher than what has been reported previously, with mean concentrations for femoral head and neck of 1.21 and 1.63 mg kg^−1^, respectively. Interestingly, the range and mean value of Cu in the IVD tissue was almost twice that of bone (0.97–6.09 vs 2.71 mg kg^−1^, respectively), even after one outlier was excluded (23.64 mg kg^−1^). Given that IVD is generally an avascular compartment, Cu compounds such as ceruloplasmin and albumin should be excluded from consideration. The question arises then of Cu’s relationship to oxygen transport in a low oxygen concentration compartment. In this case, a high Cu concentration should be rather linked with collagen formation and healing. The results at any rate suggest a significant role for Cu and its compounds in the IVD tissue. As was the case with Pb, the IVD is a more stable compartment for Cu detection compared to bone.

Zinc and Mg showed no deviation from previous findings. The biological role of magnesium ions is quite extensive [17]: they take part in nucleic acid chemistry with DNA and RNA synthesis; an array of enzymes requires their presence as the reaction co-factor; and they have a role in energetic nucleotide formation (ATP as the chelate with Mg ion). Magnesium also plays a role in the active transport of calcium and potassium ions across cell membranes, a process that is important to nerve impulse conduction, muscle contraction, and normal heart rhythm. Magnesium levels are well documented in a variety of tissues, including IVD and the similar temporomandibular joint disc [34]. Tohno et al. [4] have reported Mg at almost all levels of the spine, with an average Mg content of 1,196 mg kg^−1^, ranging from 600 to 2,200 mg kg^−1^, in agreement with our results of 758.17 mg kg^−1^ (range 182.6–2,132 mg kg^−1^ dw). Differing slightly from our findings are values for the temporomandibular joint disc [34] reported by Takano et al. of 524.74 vs 758.17 mg kg^−1^ dw, respectively. Similar values for Mg were also reported in the posterior longitudinal ligaments of the cervical spine (445 mg kg^−1^) [35], which also were less than our results (161 and 494.8 mg kg^−1^ of SD, respectively). In soft tissue of the stomach, the concentration seems to be the lowest compared to tissues described in the literature, ranging from 30 to 300 mg kg^−1^ dw [36]. Compared to IVD, the average concentration of Mg in bones may be more than two times higher at 1,792.9 mg kg^−1^ [26]. Our study confirms the literature data for bone and IVD performed separately. Magnesium concentration was approximately two times higher in bone (1,661.21 and 1,458.49 mg kg^−1^ in femoral neck and head, respectively) compared to disc (758.17 mg kg^−1^).

The majority of Mg ions are intracellular (39%), and only 1% is stored extracellularly [37], which might lead to an explanation of these tissue differences based on their cell density characteristics. The literature on their relative cellularity is unclear. Cellularity of the bone ranges from 0.5 to 10 kcells mm^−3^ [38], but we have to recall that bone is divided into two major compartments, the cortical and trabecular bone. An additional variable is the age of the patient. For the femoral head and neck, values for marrow cellularity in the newborn are 100% but fall to 60% in the 10-year-old and to 25% in the adult [39,40]. The cell density of IVD used in analytical calculations is 9 kcells mm^−3^ for annulus fibrosus, 4 kcells mm^−3^ for nucleus pulposus, and 15 kcells mm^−3^ for the cartilage end plate [41]. Other sources cite that in extreme cases, such as the cartilage, there may be ~10 cells mm^−3^ [42]. Considering the above, it is possible to infer that cellularity of the bone is double that of the disc and that the twofold higher concentration of Mg in the analyzed samples might relate to cell concentration.

Zinc, as well as Mg, is an enzyme co-factor [43] and involved in DNA and protein synthesis and cell division [44], as well as intracellular regulation [45]. Zinc is believed to have antioxidant properties with anti-aging effects and an influence on the healing process [46]. It is also believed to affect immune response mechanisms [47]. The most important role of Zn in the IVD is probably the formation the matrix metalloproteinases that are Zn-dependent endopeptidases. The metal ions act as co-factors, distinguishing these endopeptidases from the others and commonly occur both in invertebrates and plants. The role of the metalloproteinases is degradation of the extracellular matrix, and its presence or a synthesis imbalance is related to IVD degeneration [48]. It is known that Zn deficiency co-occurring in pinealectomized chickens can lead to developmental spine deformity [49]. But the pathology can not be attributed to the pathological changes in the IVD.

The close relationship of the Mg and Zn may favor a concept of simultaneous regeneration and degeneration processes taking place in the IVD. Zinc concentration in the IVD determined in our study was similar to levels encountered in posterior longitudinal ligaments by Kumai et al. (34.61 and 36 mg kg^−1^ dw, respectively) [50]. The average concentration in cartilage and bone was twice as high at 88.3 mg kg^−1^ (range 54.3–163.8 mg kg^−1^) dw and 84.58 mg kg^−1^ (SD 17.68 mg kg^−1^), respectively [26,33]. In our study, Zn concentration, similar to the findings above, was approximately twice as high in bone compared to disc. Because of a lack of reference data for Zn in non-degenerated IVD, it is not possible to state whether we should link the concentration of the element with the biological activity of matrix metalloproteinases. Based on the concentration observed in the tendons, we would be prone to expect higher Zn values in degenerated disc tissue.

The only study comparing metal concentrations in both bone and IVD was performed by Minami et al. [5] evaluating platinum levels in cis-platinum-treated patients. In that work, they showed that IVD concentration may be up to 4.3 times higher compared to bone. They determined the bone level in the vertebral body, which is the direct transportation route to and from the IVD. The concentration ratio of specific TE in bone and IVD may be related not only to exposure but also to tissue affinity and metabolic profile, which could be a basis for further studies.

## Conclusions

Except for Cu, the TE concentrations were higher in bone compared to IVD.

This study showed a higher concentration of Cu in disc tissue compared to bone, which may be related to cross-linking in collagen formation and healing processes and should be a subject of further study.

In addition, IVD tissue seems to be a more stable compartment for evaluating TE concentration, especially environmentally related TEs. In the case of disc tissue, a higher ratio of IVD samples had a concentration of Pb, Mo, and Ni within the detection threshold compared to bone. It may be better to consider IVD, compared to bone, as the indicator tissue in biochemical studies.

## Abbreviations

TE: trace elements
IVD: intervertebral disc
GF-AAS: graphite furnace atomic absorption spectrometry
DDD: degenerative disc disease
OA: osteoarthritis of the hip joint
dw: dry weight
F-AAS: flame atomic absorption spectrometry
SD: standard deviation
LOD: level of detection
DNA: Deoxyribonucleic acid
RNA: Ribonucleic acid
ATP: Adenosine triphosphate
